# Moderate levels of dietary arachidonic acid reduced lipid accumulation and tended to inhibit cell cycle progression in the liver of Japanese seabass *Lateolabrax japonicus*

**DOI:** 10.1038/s41598-018-28867-z

**Published:** 2018-07-16

**Authors:** Houguo Xu, Chengqiang Wang, Yuanqin Zhang, Yuliang Wei, Mengqing Liang

**Affiliations:** 1 Yellow Sea Fisheries Research Institute, Chinese Academy of Fishery Sciences, 106 Nanjing Road, Qingdao, 266071 Shandong China; 2 Laboratory for Marine Fisheries Science and Food Production Processes, Qingdao National Laboratory for Marine Science and Technology, 1 Wenhai Road, Qingdao, 266237 Shandong China

## Abstract

To investigate the physiological roles of dietary arachidonic acid (ARA) in fish, a feeding trial with Japanese seabass was conducted, followed by a hepatic transcriptome assay. Six experimental diets differing basically in ARA level (0.05%, 0.22%, 0.37%, 0.60%, 1.38% and 2.32% of dry matter) were used in the feeding trial. Liver samples from fish fed diets with 0.05% and 0.37% ARA were subjected to transcriptomic assay, generating a total of 139 differently expressed unigenes, which were primarily enriched in lipid metabolism and cell cycle-related signaling pathways. Then, qRT-PCR validation on lipid metabolism and cell cycle-related genes as well as corresponding enzyme-linked immunosorbent assay of selected proteins were conducted with liver samples from all six groups. Moderated ARA levels reduced lipogenesis and stimulated β-oxidation concurrently, but high ARA levels seemed to affect lipid metabolism in complicated ways. Both gene expression and protein concentration of cell cycle-related proteins were decreased by moderate levels of dietary ARA. The lipid content and fatty acid composition in fish confirmed the transcription and protein concentration results related to lipid metabolism. In conclusion, moderate levels of dietary ARA (0.37% and 0.60%) reduced lipid accumulation and tended to inhibit cell cycle progression in the liver of Japanese seabass.

## Introduction

Arachidonic acid (ARA, C20:4n-6), as an n-6 long chain-polyunsaturated fatty acid (LC-PUFA), has been demonstrated to be an essential fatty acid for marine fish^1^. In the past 20 years, special attention has been paid to the physiological roles of ARA in fish. Dietary ARA has been reported to be able to regulate a series of fish physiological processes such as growth, survival, stress resistance, immunity, and reproduction^2–5^. In our previous studies with Japanese seabass, we have investigated the effects of dietary ARA on growth performances, non-specific immunity, as well as gene expressions of fatty acid-binding proteins (FABP) and fatty acid transport proteins (FATP) in various tissues^6–8^. With another marine fish species tongue sole (*Cynoglossus semilaevis*), we also investigated the influence of dietary ARA on the gonadal steroidogenesis^9^. To more comprehensively understand the roles of ARA in fish physiology, transcriptomic assay was used in the present study to screen the responsive metabolic pathways in Japanese seabass to dietary ARA supplementation.

With the rapid development of high-throughput sequencing technology, transcriptomic analysis has been a useful and efficient tool in investigating the metabolic responses of animals to experimental treatments at the gene expression level. In the present study, liver samples from two out of six experimental groups with graded dietary ARA levels, that is, the group without extra ARA supplementation and the group with a suitable ARA level in terms of growth performances^6^, were subjected to the transcriptomic assay. The screened responsive metabolic pathways to dietary ARA from the transcriptomic assay, that is lipid metabolism and cell cycle-related pathways, were then investigated in all the six experimental groups.

The modulation of lipid metabolism by ARA has been well documented in studies with terrestrial animal^10–15^. In human and mammals, ARA has been widely observed to reduce the triacylglycerol (TG) accumulation in liver, plasma and adipose tissue, and this regulation could be via both the ARA-derived eicosanoids^13,15,16^ and non-eicosanoid ways such as directly binding to DNA thereby interfering with gene expression or chromosomal structure^17^. However, in fish, little information has been reported about the roles of dietary ARA in lipid metabolism, especially lipid accumulation which influences fish product quality and thus is a very important concern of fish farming. The effects of dietary ARA on lipid accumulation in fish has been considered in only two previous studies, and both studies indicated the potential of ARA in modulating lipid accumulation^18,19^. Therefore, investigation on effects of dietary ARA on lipid metabolism in Japanese seabass in the present study would provide new data in this research area. Besides lipid metabolism, in the present study it was the first time to observe the regulation of cell cycle by dietary ARA in fish. Regulation of cell cycle progression by ARA has been reported in a number of mammal studies including *in vitro* studies^20–28^, but some contradictory results existed. Investigation of the regulatory effects of ARA on cell cycle in fish could be helpful to comparison of ARA effects on cell cycle progression among different animal species.

Japanese seabass is one of the most commercially valuable aquaculture species in Asia. It is carnivorous and has the ability to adapt to a wide range of salinity. The repaid growth and relatively high lipid deposition in tissues makes this fish also a good model for studies on fatty acid and lipid nutrition. With Japanese seabass cultured in seawater, we have investigated the nutritional effects of a series of fatty acids and lipid sources such as DHA, EPA, ARA, α-linolenic acid (LNA), oleic acid (OA), steric acid (SA), palmic acid (PA), steric acid (SA), medium chain-fatty acid (MCA), fish oil, soybean oil, and linseed oil^6,29–31^. Genetic properties of some fatty acid/lipid metabolism-related proteins such as Δ6 fatty acid desaturase (FADS2), sterol-regulatory element binding proteins (SREBP), peroxisome proliferator-activated receptor (PPAR), FABP, and FATP^7,8,32,33^, as well as their response to different dietary fatty acids/lipids have also been investigated in these studies. As a following-up study, the present study is aimed at investigating the potential expanded effects of dietary ARA on physiological processes of Japanese seabass, with a hepatic transcriptome assay. The results will provide useful information for better understanding the physiological roles of ARA in fish.

## Results

### Sequence assembly and annotation of unigenes

In this study, three pooled liver RNA samples were prepared for each dietary group (ARA-0.05 and ARA-0.37). Six cDNA libraries were then constructed to perform Illumina sequencing. A total of 155,815,580 and 165,185,278 clean reads were obtained for groups ARA-0.05 and ARA-0.37 respectively, giving rise to total clean bases of 23.37 and 24.77 G, respectively (see Supplementary Table S1). The average Q20 and Q30 (the sequencing error rate 1% and 0.1% respectively) of the experimental samples was 96.59% and 90.58% respectively, indicating the high accuracy of the sequencing processes. Raw reads were deposited at the National Center for Biotechnology Information (NCBI)’s Sequence Read Archive under Accession No. SRP107356 (ARA_C and ARA_L in the archived data represents ARA-0.05 and ARA-0.37 respectively).

The reads produced were used for de novo assembly. A total of 261,947 transcripts and 191,857 unigenes were obtained, of which 73,802 transcripts and 29,414 unigenes were >1000 bp (see Supplementary Table S2 and Supplementary Figs S1 and S2). The minimum, mean, and maximum length of ugnigenes was 201, 680, and 19,863 bp respectively (see Supplementary Fig. S2). The unigenes were subjected to annotation by matching sequences against Databases NCBI non-redundant protein sequences (Nr), NCBI non-redundant nucleotide sequences (Nt), Protein family (Pfam), Clusters of Orthologous Groups of proteins (KOG/COG), Swiss-Prot, KEGG Ortholog database (KO), and Gene Ontology (GO) using BLAST searching with an E value of 1 × 10^−5^, 1 × 10^−5^, 0.01, 1 × 10^−3^, 1 × 10^−5^, 1 × 10^−10^, and 1 × 10^−6^ respectively. Of the total unigenes, 24.33% was matched in the Nr database; 34.7% matched in Nt; 19.67% matched in Pfam; 11.23% matched in KOG/COG; 20.08% matched in Swiss-Prot; 13.24% matched in KO; and 19.78% matched in GO. 6.67% of the total unigenes were annotated in all databases and 42.55% were annotated in at least one database (see Supplementary Table S3 and Supplementary Figs S3 and S4).

### Differentially expressed genes (DEGs) between groups ARA-0.05 and ARA-0.37

**Figure 1:**
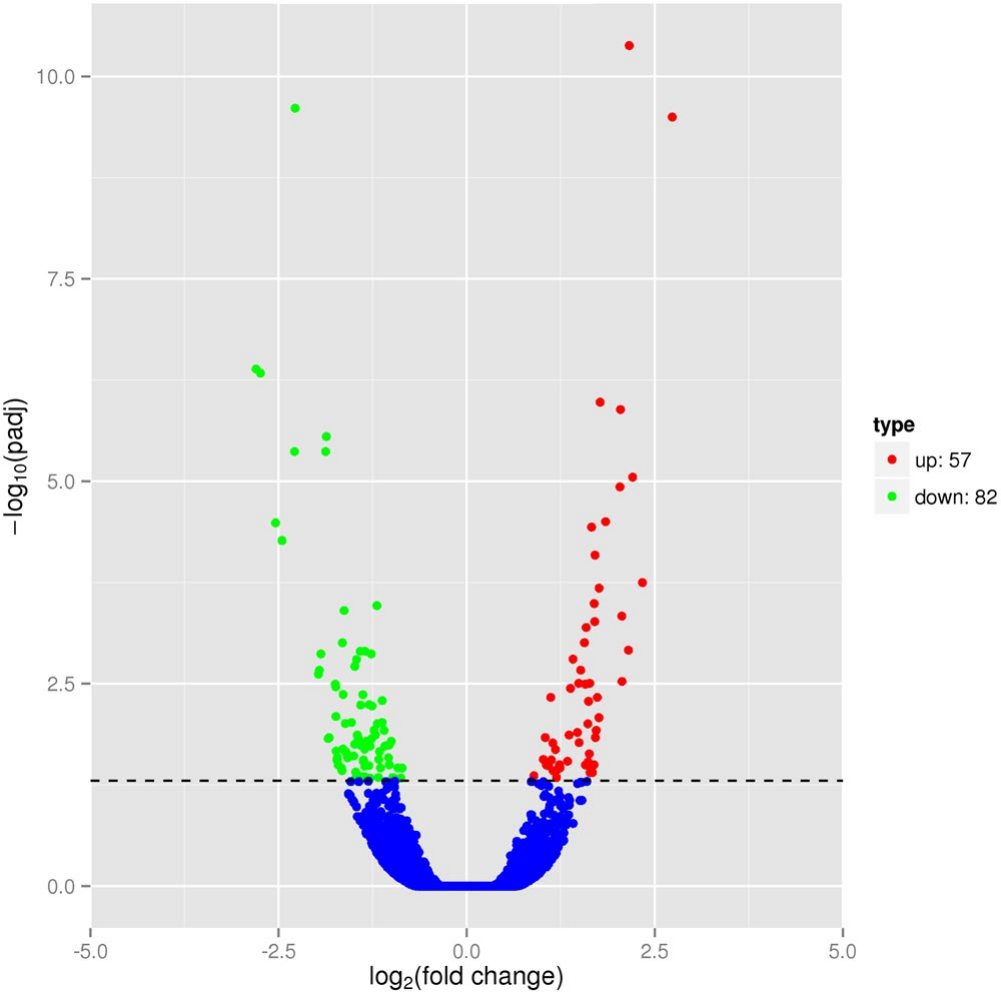
Volcano plot of differentially expressed genes.

A total of 139 unigenes were differentially expressed (adjusted *P* value < 0.05) between groups ARA-0.05 and ARA-0.37 (Fig. 1). Compared to the diet without ARA supplementation, ARA supplementation at the level of 0.37% (of dry matter) up-regulated transcription of 57 genes and down-regulated those of 82 genes. To gain insights into the biological processes regulated by the dietary treatments, the DEGs were subjected to GO functional enrichment and KEGG pathways enrichment analysis. Although the DEGs were enriched in no GO term or KEGG pathway at a significant level, the KEGG pathway enrichment was still biologically indicative to some extent. The KEGG pathway enrichment analysis showed that PPAR signaling pathway, AMPK signaling pathway, cell cycle, and oocyte meiosis may be the primary responsive biological processes to dietary ARA. This analysis, along with the detailed DEG list, indicated that lipid metabolism and cell cycle-related processes may be the main target processes of ARA effects. Lipid metabolism and cell cycle-related DEGs and 6 more selected DEGs with fold changes >4 were listed in Table 1.

**Table 1:** Part of differentially (*P* < 0.01) expressed genes between groups ARA-0.05 and ARA-0.37.

Descrption	Featured ID	Log_2_FC	Adjusted *P*
*Lipid metabolism*			
Stearoyl-CoA desaturase 1 (SCD1)	c74613_g1	−2.28↓	<0.001
Apolipoprotein E (ApoE)	c70457_g2	−1.65↓	<0.001
Carnitine O-palmitoyltransferase 1α (CPT-1α)	c77836_g1	1.57↑	<0.001
Fatty acid-binding protein 1 (FABP1)	c74726_g2	1.24↑	0.035
Phosphoenolpyruvate carboxykinase (PEPCK)	c65493_g1	2.21↑	<0.001
Glycogen synthase (GS)	c74396_g2	−1.87↓	<0.001
*Cell cycle*			
G2/mitotic-specific cyclin-B1-like (Cyclin B1)	c69131_g1	−1.97↓	0.002
Cyclin-dependent kinase 1 (Cdc2)	c39940_g1	−1.61↓	0.010
Myc proto-oncogene protein (MYC)	c53294_g1	1.61↑	0.010
Cell division cycle protein 20 homolog (Cdc20)	c66735_g1	−1.58↓	0.026
Aurora kinase A (AURKA)	c70175_g1	−1.72↓	0.028
Cyclin I	c69940_g1	−1.28↓	0.046
*Other DEGs with FC* > 4			
Small integral membrane protein 1 (SMIM1)	c65481_g1	−2.7983↓	<0.001
Large neutral amino acids transporter (LAT)	c72167_g1	−2.739↓	<0.001
Cytoskeleton-associated protein 2-like (CSAP2)	c68538_g1	−2.2877↓	<0.001
Creatine kinase M-type (CKM)	c75778_g2	−2.5395↓	<0.001
Tensin-like C1 domain-containing phosphatase (TENC1)	c64699_g1	−2.4533↓	<0.001
Zinc finger and BTB domain-containing protein 16 (ZBTB16)	c59375_g1	2.1504↑	0.001

“↑” and “↓” represents the up- and down-regulated genes in group ARA-0.37 respectively; FC, fold change.

### Validation of the DEG results by qRT-PCR

**Figure 2:**
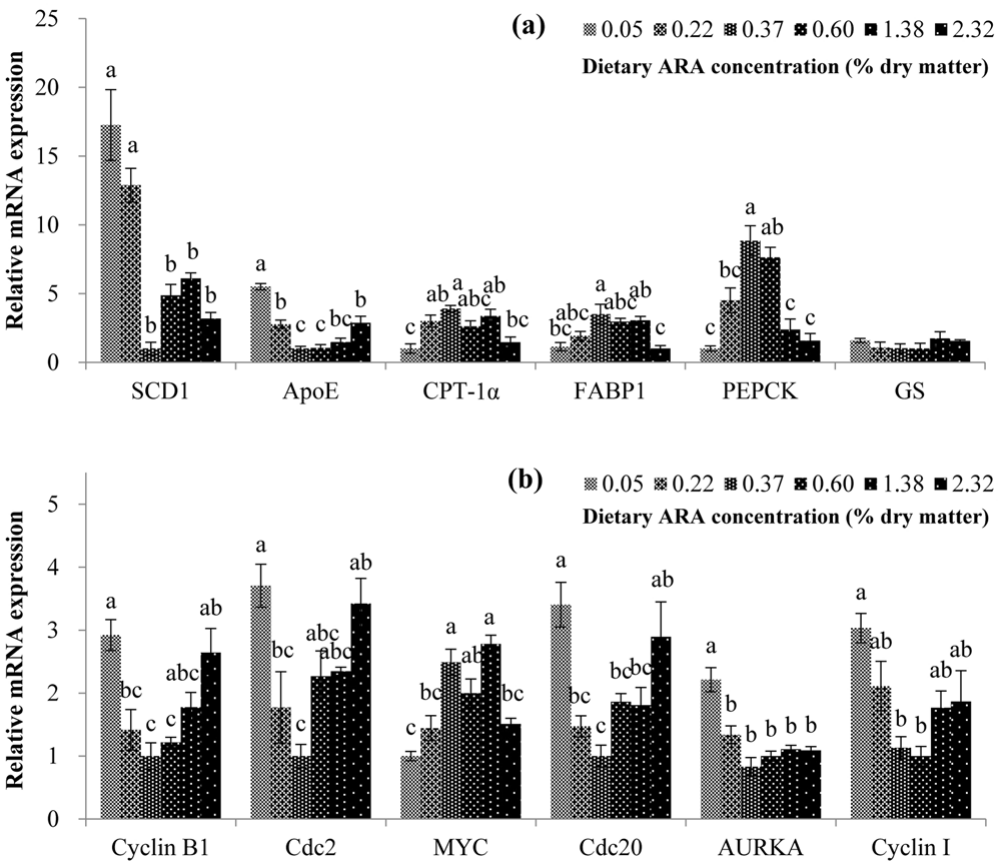
Validation of the transcriptome results by qRT-PCR measurement. (**a**) Lipid metabolism-related genes. (**b**) Cell cycle-related genes. The mRNA levels were expressed relative to β-actin. Results are expressed as means ± standard error (n = 3). Different letters above the bars denote significant (*P* < 0.05) differences among dietary groups.

To confirm the DEG results from the transcriptomic assay, as well as to gain insights into the regulatory effects of different dietary ARA levels, lipid metabolism and cell cycle-related DEGs were validated with qRT-PCR in all the six experimental groups (Fig. 2). The results showed that for most of these genes (11 out of 12) the qRT-PCR results were in good agreement with the transcriptomic results, although the fold change might be different to some extent between the two sets of results. This confirmed the authenticity of the illumina sequencing results. A conflicting result was observed in gene expression of glycogen synthase (GS) which was significantly (*P* < 0.05) influenced by dietary ARA in the transcriptomic result but not in the qRT-PCR result. The qRT-PCR results among all the six dietary groups also showed that dietary ARA regulated the DEGs in dose dependent manners. Compared to the group without ARA supplementation (ARA-0.05), moderate ARA supplementation (0.37~1.38%) enhanced the mRNA expression of carnitine O-palmitoyltransferase-1α (CPT-1α), FABP1, phosphoenolpyruvate carboxykinase (PEPCK), and myc proto-oncogene protein (MYC), but reduced the mRNA expression of apolipoprotein E (ApoE), G2/mitotic-specific cyclin-B1 (Cyclin B1), cyclin-dependent kinase 1 (CDK1, Cdc2), Cell division cycle protein 20 (Cdc20), and Cyclin I; however, the highest ARA level (2.32%) exerted no significant influence (*P* > 0.05). Dietary ARA supplementation significantly (*P* < 0.05) down-regulated the transcription of aurora kinase A (AURKA). Dietary ARA at levels of 0.37~2.32% (*P* < 0.05) led to significantly (*P* < 0.05) lower transcription of stearoyl-CoA desaturase 1 (SCD1) than ARA levels of 0.05% and 0.22%. The mRNA expression of SCD1 in group ARA-0.05 was 17 times that in group ARA-0.37.

**Figure 3:**
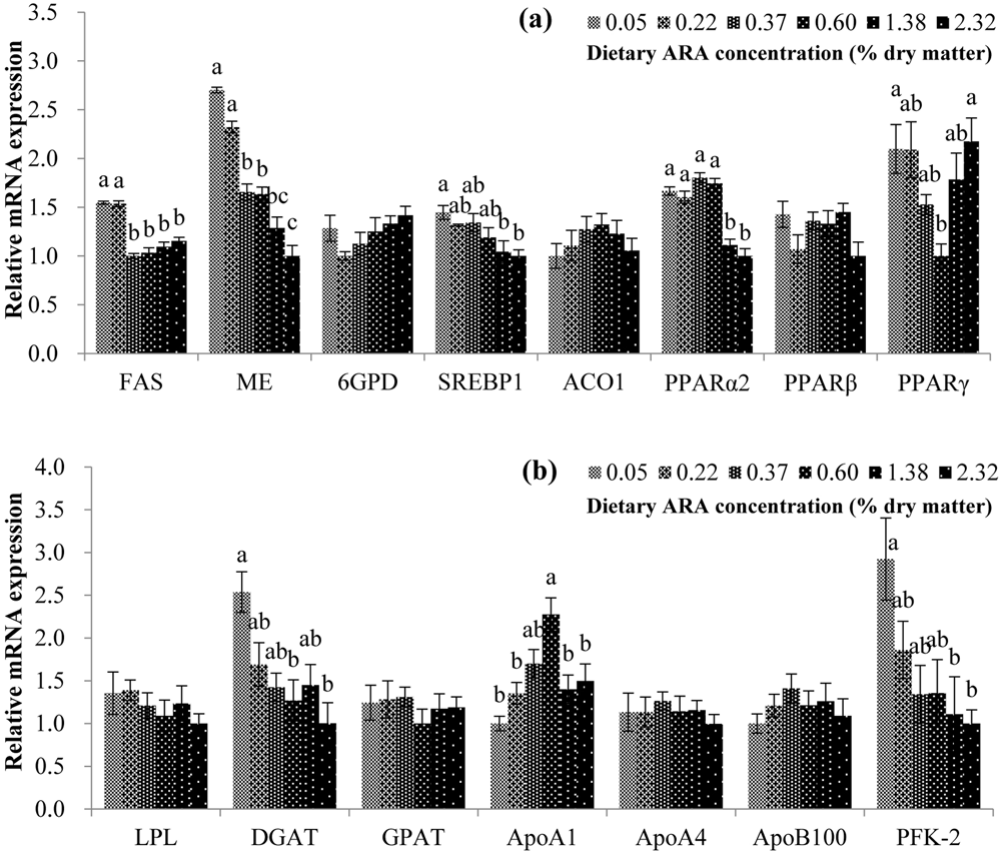
Relative mRNA expression of lipid metabolism-related genes in the liver of experimental fish. (**a**) Lipogenesis and β-oxidation-related genes; (**b**) Other lipid metabolism-related genes. The mRNA levels were expressed relative to β-actin. Results are expressed as means ± standard error (n = 3). Different letters above the bars denote significant (*P* < 0.05) differences among dietary groups.

To get a better understanding of the regulation of lipid metabolism-related gene expression by dietary ARA, transcription of 15 more lipid metabolism-related genes, which were not significantly (*P* > 0.05) different between groups ARA-0.05 and ARA-0.37 in the transcriptomic result, were also assayed by qRT-PCR in all the six experimental groups (Fig. 3). The results showed that although gene expressions of lipoprotein lipase (LPL), glucose-6-phosphate dehydrogenase (6GPD), phosphoribosyl pyrophosphate transamidase (GPAT), acyl-CoA oxidase 1 (ACO1), PPARβ, apolipoprotein A4 (ApoA4), and apolipoprotein B100 (ApoB100) were not significantly (*P* > 0.05) different among dietary groups, other genes showed significant (*P* < 0.05) difference. Higher levels of dietary ARA (0.37~2.32%) reduced the gene expression of fatty acid synthetase (FAS) and PPARα2, while transcription of malic enzyme (ME), SREBP1, diacylglycerol acyl transferase (DGAT), and 6-phosphofructo-2-kinase (PFK-2) decreased significantly (*P* < 0.05) with increasing dietary ARA levels.

Group ARA-0.37 had significantly (*P* < 0.05) higher (*P* < 0.05) gene expression of PPARγ than groups ARA-0.22 and ARA-2.32, while group ARA-0.60 had significantly higher (*P* < 0.05) gene expression of apolipoprotein A1 (ApoA1) than other groups except group ARA-0.37. Another interesting observation from these results was that although the transcriptomic results showed no significant (*P* > 0.05) difference in transcriptions of FAS and ME between groups ARA-0.05 and ARA-0.37, significant (*P* < 0.05) differences were observed in the qRT-PCR assay. One explanation is that in the qRT-PCR assay the within-group errors were controlled to be at lower levels (see Supplementary Fig. S5).

### Hepatic protein concentration corresponding to transcription results

**Figure 4:**
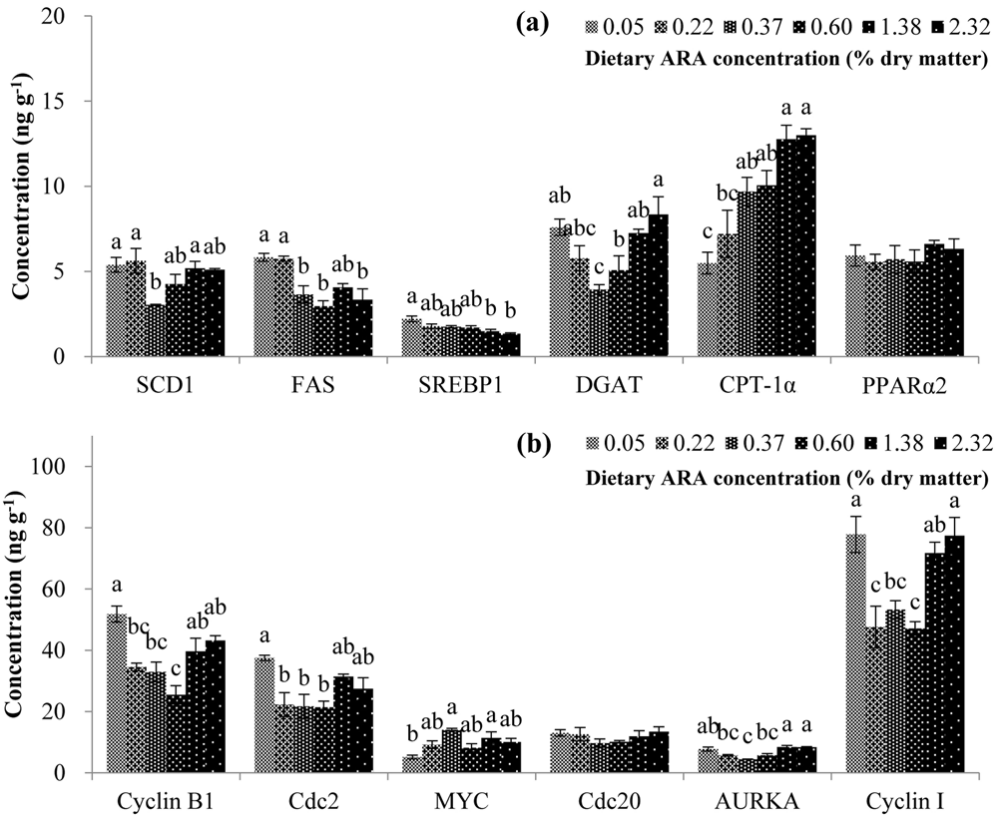
Hepatic protein concentration of selected proteins assayed with ELISA methods. (**a**) Lipid metabolism-related proteins; (**b**) Cell cycle-related proteins. Results are expressed as means ± standard error (n = 3). Different letters above the bars denote significant (*P* < 0.05) differences among dietary groups. The concentration of SREBP1 was expressed as μg g^−1^ liver tissue.

To investigate the regulatory effects of ARA at post-transcriptional levels, concentration of selected proteins, i.e., 6 lipid metabolism-related proteins and 6 cell cycle-related proteins, in liver samples from all the six experimental groups was assayed with enzyme-linked immunosorbent assay (ELISA) method (Fig. 4). The protein concentration results were generally consistent with the gene expression results in most proteins. However, inconsistent results still existed in some proteins, especially in groups with high levels of dietary ARA supplementation. Different from the gene expression results, with increasing levels of dietary ARA, the concentration of CPT-1α significantly increased, but the concentration of DGAT first decreased (from ARA-0.05 to ARA-0.37) and then increased (from ARA-0.37 to ARA-2.32). Despite of the significant regulation of PPARα2 gene expression by dietary ARA, no significant difference was observed in protein concentration of PPARα2 among different dietary groups. For cell cycle-related proteins, obvious difference between gene expression results and protein concentration results was observed in Cdc20 and AURKA. Despite of significant difference in Cdc20 gene expression among dietary groups, no significant difference in protein concentration of Cdc20 was observed among dietary groups. Different from the gene expression result, the highest protein concentration of AURKA was observed in groups ARA-1.38 and ARA-2.32, which were significantly higher compared to groups ARA-0.22, ARA-0.37, and ARA-0.60.

### Lipid accumulation and fatty acid profiles of fish tissues

**Figure 5:**
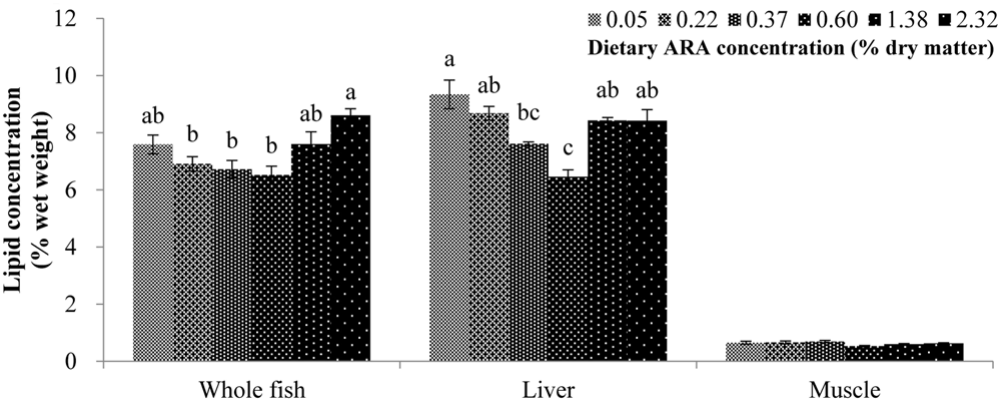
Lipid contents in experimental fish. Results are expressed as means ± standard error (n = 3). Different letters above the bars denote significant (*P* < 0.05) differences among dietary groups.

Since the lipid metabolism-related genes was greatly influenced by dietary ARA, lipid metabolism-related phenotypic performances, the lipid (Fig. 5) and fatty acid (Table 2) compositions of experimental fish, were also assayed to confirm the regulatory effects of dietary ARA on lipid metabolism. At the end of the feeding trial, the average lipid contents of fish slightly increased compared to the average initial ones in whole fish body (7.33% vs 7.08% of wet weight) as well as in fish tissues, liver (8.16% vs 7.95%) and muscle (0.63% vs 0.61%). At the end, fish from groups ARA-0.37, ARA-0.60 and ARA-1.38 had significantly (*P* < 0.05) lower body lipid content than fish from group ARA-2.32. The lowest liver lipid content was observed in group ARA-0.60, significantly (*P* < 0.05) lower than those in other groups except group ARA-0.37, while the highest value was observed in group ARA-0.05, significantly (*P* < 0.05) higher compared to groups ARA-0.37 and ARA-0.60. No significant (*P* > 0.05) difference was observed in muscle lipid content among dietary groups (*P* > 0.05).

**Table 2:** Fatty acid profiles of liver and muscle of the experimental fish (% total fatty acid, means ± standard error, n = 3).

Fatty acids	ARA-0.05	ARA-0.22	ARA-0.37	ARA-0.60	ARA-1.38	ARA-2.32
*Liver*						
C18:0	5.02 ± 0.50	5.96 ± 0.16	5.64 ± 0.42	6.17 ± 0.58	5.14 ± 0.23	6.10 ± 0.31
ΣSFA	28.54 ± 1.26^a^	29.52 ± 0.82^a^	26.54 ± 0.95^a,b^	26.44 ± 1.15^a,b^	22.36 ± 0.40^b^	23.78 ± 2.32^a,b^
C18:1n-9	24.90 ± 1.97^a^	24.35 ± 4.82^a^	23.67 ± 3.15^a,b^	19.58 ± 1.76^a,b^	15.53 ± 0.67^a,b^	11.80 ± 1.98^b^
∑MUFA	35.36 ± 2.46^a^	34.87 ± 5.34^a^	33.68 ± 4.02^a^	27.56 ± 2.20^a,b^	21.95 ± 0.54^a,b^	16.5 ± 2.73^b^
C20:4n-6	1.14 ± 0.09^d^	2.90 ± 0.65^c^	3.52 ± 0.27^b^	7.15 ± 0.64^a^	10.84 ± 0.62^a^	17.33 ± 0.64^a^
∑n-6 PUFA	12.09 ± 0.78^c^	12.55 ± 2.12^c^	13.78 ± 0.95^c^	16.92 ± 1.28^b,c^	21.46 ± 0.94^b^	28.21 ± 1.30^a^
C20:5n-3	2.06 ± 0.19	1.63 ± 0.44	1.93 ± 0.57	2.19 ± 0.20	2.57 ± 0.42	2.04 ± 0.25
C22:6n-3	13.26 ± 1.53	12.31 ± 1.77	14.50 ± 1.85	17.35 ± 1.40	18.95 ± 0.05	16.02 ± 1.57
∑n-3 PUFA	17.87 ± 1.83	15.96 ± 2.56	18.82 ± 2.82	21.89 ± 1.73	24.41 ± 0.38	20.20 ± 1.97
*Muscle*						
C18:0	8.03 ± 0.31	7.71 ± 0.11	7.66 ± 0.34	8.14 ± 0.17	7.98 ± 0.13	7.92 ± 0.27
ΣSFA	32.30 ± 0.82^a^	30.44 ± 0.07^a,b^	30.05 ± 0.64^a,b,c^	30.23 ± 0.48^a,b,c^	28.83 ± 0.12^b,c^	27.67 ± 0.78^c^
C18:1n-9	18.00 ± 0.44^a^	17.82 ± 0.93^a^	16.31 ± 0.40^a,b^	13.58 ± 0.11^b,c^	13.12 ± 1.00^b,c^	11.41 ± 0.72^c^
∑MUFA	24.99 ± 0.47^a^	24.56 ± 1.14^a^	22.37 ± 0.70^b,c^	18.12 ± 0.10^c,d^	18.05 ± 1.35^c,d^	15.36 ± 1.14^d^
C20:4n-6	2.93 ± 0.22^e^	4.53 ± 0.15d^e^	5.90 ± 0.45^d^	10.73 ± 0.22^c^	13.74 ± 0.84^b^	21.86 ± 0.45^a^
∑n-6 PUFA	11.66 ± 0.35^d^	12.71 ± 0.19^d^	13.64 ± 0.25^d^	17.39 ± 0.55^c^	22.84 ± 0.85^b^	29.52 ± 1.14^a^
C20:5n-3	3.27 ± 0.08^a^	2.63 ± 0.15^b^	2.55 ± 0.20^b^	2.32 ± 0.06^b,c^	2.36 ± 0.08^b,c^	1.80 ± 0.14^c^
C22:6n-3	18.87 ± 0.45^a^	17.18 ± 0.57^a^	18.90 ± 0.90^a^	19.18 ± 0.88^a^	15.81 ± 0.79^a,b^	12.98 ± 0.97^b^
∑n-3 PUFA	24.41 ± 0.43^a^	21.86 ± 0.71^a,b^	23.53 ± 0.73^a,b^	23.38 ± 0.90^a,b^	20.13 ± 0.77^b^	16.33 ± 0.85^c^

Values in the same row with no common superscript letters are significantly different (*P* < 0.05). SFA, saturated fatty acid; MUFA, monounsaturated fatty acid; PUFA, polyunsaturated fatty acid.

The fatty acid profiles of fish liver and muscle reflected closely those of the diets. With increasing dietary ARA levels, the ARA contents increased significantly (*P* > 0.05) while the contents of saturated fatty acids (SFA) and monounsaturated fatty acid (MUFA) decreased significantly (*P* > 0.05). The contents of EPA, docosapentaenoic acid (DPA, C22:5n-3), and DHA in muscle but not in liver were reversely related to ARA contents. Liver had a higher average MUFA content but lower SFA and ARA contents than muscle.

## Discussion

Due to the rapid development of sequencing technologies, high quality transcriptomic assay has been widely used in biological studies. It provides an efficient way to investigate the genetic properties of experimental animals. Japanese seabass is an important fish species for both aquaculture industry and academic research. In the present transcriptomic assay with Japanese seabass liver, a total of 48.14 G clean bases data and 191,857 unigenes were generated, greatly enriching the transcriptome data of *L. japonicus* and prompting the genome studies of teleost. In a recently published RNA-Seq data with kidney, liver, and spleen of Japanese seabass, 101,860 unigenes were obtained and 29.59% of them (30,142) were annotated in the public databases^34^. The advance in amount of generated unigenes in the present study may be partly attributed to the high sequencing quality. Moreover, in the present study 42.55% of total unigenes (81,635) were annotated in seven public databases. The application of more databases (7 in the present study vs 4 in the previous study) may contribute to the increase in amount of annotated unigenes. Nevertheless, there could be still a large amount of novel genes, which need to be confirmed by further studies. The GO and KEGG classification analysis of the annotated unigenes showed that of the GO annotated unigenes the “biological process” category was dominant, and in KEGG classification a dominant “metabolism” category was obtained. Similar results were also observed in other transcriptomic assays with fish liver, and these results conformed to the physiological roles of fish liver, which is a metabolism center of fish and plays vital roles in a wide range of biological processes^35–37^.

Besides enhancing the understanding of genetic properties of experimental animals, transcriptomic assay is also useful in screening the target physiological processes of experimental treatments. In the present study, a total of 139 unigenes were differentially expressed between the two groups subjected to transcriptomic assay, that is, the group without extra ARA supplementation (containing 0.05% ARA) and the one with suitable ARA dietary ARA content (0.37%) in terms of growth performances. The screened responsive genes to dietary ARA greatly enhanced our understanding of the effects of ARA on gene expressions in fish. They were new evidence for the regulatory effects of ARA on lipid metabolism-related genes, which were observed mostly in terrestrial animals and human^10–15^. The results also indicated that PPAR signaling pathway and AMPK signaling pathway were possibly involved in the regulatory effects. More interestingly, it was the first time in fish to observe that dietary ARA regulated a series of cell cycle-related genes such as Cyclin B1, Cdc2, MYC, Cdc20, AURKA, and Cyclin I.

The assay of the screened DEGs in all the six groups with graded ARA levels provided a more comprehensive understanding of the regulatory effects of different ARA levels. The effects of dietary ARA on most DEGs seemed to be dose-dependent, as has been observed in previous studies^6,38,39^. For most DEGs, the efficacy of ARA was different between moderate (0.22~0.60%) and high (1.38% and 2.32%) dietary ARA levels. The imbalance between ARA and EPA and the consequent imbalance between ARA- and EPA-derived eicosanoids were assumed to contribute to the ineffectiveness or even inhibitory effects of excess ARA^40^.

Among the lipid metabolism-related DEGs, the most significantly affected gene was SCD1. Dietary ARA at levels of >0.22% considerably lowered the SCD1 transcription compared to the groups with 0.05% or 0.22% dietary ARA. The protein concentration of SCD1 in liver of group ARA-0.37 was also significantly lower compared to group ARA-0.05 and ARA-0.22. The inhibition of SCD activity and gene expression by ARA has been reported in human and mammal studies^16,41,42^. ARA may regulate SCD through a non-eicosanoid way, that is binding to DNA thereby controlling gene expression or inducing chromosomal abberations, or a 5-hydroxy-eicosatetraenoicacid (5-HETE) mediated way^17^. Although ARA has also been reported to inhibit the synthesis of LC-PUFA by inhibiting Δ6 fatty acid desaturase^18,42^, it was assumed that it was the inhibition of SCD which resulted in less MUFA being stored in TG, cholesterol ester, and phospholipids pools, and consequent less lipid accumulation in animal body^43^.

In human and mammals, along with the inhibition of fatty acid desaturases, ARA has been widely observed to reduce the TG accumulation in liver, plasma and adipose tissue, and this regulation was partly via the ARA-derived eicosanoids^13,15,16^. However, contradictory results have also been observed. In several studies, ARA increased or did not influence the hepatic TG and total lipid^12,44^, while ARA-derived prostaglandins (PG) enhanced the adipogenesis^14,45^. In adipose tissues from obese humans, ARA or PGs stimulated the leptin release^10^, but another study with primary cultured rat adipocytes showed that ARA inhibited both basal and insulin stimulated leptin secretion^11^. The regulation of lipid accumulation by ARA seemed complicated. The efficacy of ARA may mainly be related to the experimental ARA dose and the target tissue. The esterification form of ARA, i.e., phospholipid, TG, or cholesteryl esters, may also affect the effects of ARA on lipogenesis and TG secretion^46,47^.

In fish, little information has been available about the regulation of lipid accumulation in fish body and tissues by ARA. In a recent study with juvenile grass carp (*Ctenopharyngodon idellus*), dietary ARA supplementation at the level of 0.3% lowered the fat content in hepatopancreas, the intraperitoneal fat ratio, and the total lipid content in whole body^18^. A previous study with gilthead bream fingerlings (*Sparus aurata*) also showed reduced lipid levels in muscle, gill, liver, and whole fish body in ARA-supplemented groups^19^. These results were in accordance with the present result about the liver lipid content, which was reduced by 0.37% and 0.60% dietary ARA. In whole fish body, although the lowest lipid content was also observed in group ARA-0.37, the highest lipid content was observed in group ARA-2.32. This confirmed again the dose-dependent characteristic of ARA effects, but it is still difficult to explain the elevation of lipid content in fish fed a very high level of dietary ARA.

Transcriptions of other lipid metabolism-related genes, as well as the corresponding protein concentration, in response to different dietary ARA levels were generally consistent with the regulation of lipid accumulation by ARA. The fatty acid synthesis-related genes such as ME and SREBP1, as well as the key enzyme in TG synthesis, DGAT, showed decreased transcription with increasing dietary ARA levels, and FAS, the rate-limiting enzyme in fatty acid synthesis, showed significantly lower gene expression in groups with 0.37~2.32% dietary ARA than in groups with 0.05% or 0.22% dietary ARA. The effects of dietary ARA on hepatic protein concentration of lipogenic proteins, FAS, SREBP1 and DGAT, generally had similar patterns with gene expression results, except that no significant difference in protein concentration of DGAT was observed between groups with low (0.05% and 0.22%) and high (1.38% and 2.32%) levels of dietary ARA. Response of lipogenic genes to dietary ARA contributed to explain the lipid lowering effects of moderate levels (0.37% and 0.60%) of dietary ARA^18,20^. However, it cannot well explain the re-rise of lipid content in groups with high dietary ARA levels (1.38% and 2.32%). Other lipid metabolism-related genes, which responded to dietary ARA differently, must also be involved in the regulation of lipid accumulation by ARA. Although PPARγ plays lipid-increasing roles mainly in adipose tissues^48^, its gene expression in liver was decreased by moderate dietary ARA levels, as also observed in other studies^18,49^. However, with further increase of dietary ARA, the liver gene expression of PPARγ thereby increased again, and this was well consistent with the lipid content results. Similar trend was also observed in hepatic protein concentration of DGAT, as mentioned above. These results indicated that compared to the control diets, diets with high levels of ARA may have no significant influence on lipogenesis. However, compared to the control, high levels of dietary ARA significantly increased the hepatic protein concentration of CPT-1α, which is a rate-limiting enzyme for β-oxidation in mitochondria, while hepatic protein concentration of PPARα2, which was a newly cloned Japanese seabass PPARα subtype in our previous studies^33^ and appeared to be mainly involved in fatty acid oxidation, was not significantly influenced by dietary ARA. The effects of high levels of dietary ARA on lipid metabolism seemed complicated, possibly being stimulative to both TG synthesis and β-oxidation, and consequently being inhibitive to fatty acid synthesis by feed-back mechanisms. In addition, inconsistent results were observed between gene expression level and protein concentration level regarding the effects of high levels of dietary ARA on β-oxidation related proteins, CPT-1α and PPARα2. Based on the present information, this result was difficult to explain. Future studies are needed to elucidate the mechanisms involved in the different regulation on gene expression level and protein translation level by excess dietary ARA.

Although marine fish have limited capacity to utilize carbohydrate^50^, in the present study effects of dietary ARA on gene expression of two carbohydrate metabolism-related enzymes correlated well with the regulation of lipid accumulation by ARA. Moderate levels of dietary ARA enhanced the gene expression of PEPCK, a key enzyme in gluconeogenesis. The reduction of lipid accumulation may partly be attributed to the increased gluconeogenesis. In addition, gene expression of PFK-2, an important enzyme in glycolysis activation, was down-regulated by increased levels of dietary ARA. This could reduce the supply of required materials for fatty acid synthesis such as pyruvic acid and NADPH.

Besides the genes related to fatty acid/lipid synthesis or oxidation, some genes linked to fatty acid transport were also influenced by dietary ARA. Dietary ARA increased the gene expression of FABP1 in a dose-dependent manner in this study. ARA and its eicosanoid metabolites are high affinity ligands of FABPs and thus could induce the expression of FABPs^51^. On the other hand, FABPs facilitates the intracellular transport of ARA^52^ and may mediate the genetic effect of ARA^53^. However, the inefficacy of excess dietary ARA in regulating FABP transcription remained inexplicable and needed to be elucidated by further studies.

Regulation of another fatty acid transport gene, FATP1 (slc27a1), which transports fatty acids across the cell membrane in liver, by ARA has also been reported. Dietary fungal oil enriched in ARA down-regulated the FATP1 gene expression in mouse liver, likely through PPARα^20,54^. In our previous studies with Japanese seabass, gene expression of a subtype of FATP1, FATP1b but not FATP1a, was also down-regulated by dietary ARA supplementation^8^. The down-regulation of FATP expression could lead to less fatty acid transported into the liver for various purposes including TG formation.

Gene expression of the apolipoproteins, ApoE and ApoA1, were also influenced by dietary ARA in the present study. Dietary ARA at 0.60% led to the highest ApoA1 gene expression while surprisingly the dietary ARA supplementation reduced the gene expression of ApoE. ApoE is involved in cholesterol and TG transport and has anti-inflammatory properties^55^. The ARA-induced decrease in ApoE gene expression could be a consequent result of decreased basic TG level in liver or a secondary effect of dietary ARA through inflammatory reactions. ApoA1 is the major apolipoprotein of plasma high density lipoproteins and it is the preferential receptor of phospholipid and free cholesterol^56^. Phospholipids even up-regulate ApoA1 synthesis^57^. The enhancement of ApoA1 transcription by ARA might be related to the preferential incorporation of ARA into phospholipids.

In the present study, the fatty acids profiles in liver and muscle were also analyzed, in order to better understand the effects of ARA on lipid accumulation. Dietary ARA was well incorporated into fish tissues, and the tissue ARA contents significantly increased with increasing dietary ARA levels. Muscle had a higher ARA content than liver. However, the muscle lipid content was not significantly influenced by ARA. This could be due to that the lipid content in the muscle was very low and it may mask the effects of ARA. With increasing ARA, the tissue MUFA contents decreased. In liver, which had higher MUFA content than muscle, the decrease in MUFA content may lead to the decrease of MUFA incorporation into liver TG, and subsequently lead to the reduction of liver lipid content. As stated previously, the reduction of MUFA content in liver by ARA was probably mainly attributed to the down-regulation of SCD by ARA rather than the decrease of C18:0 in diets, which is the substrate of SCD. Basically, C18:0 is relatively unimportant to fish, and dietary C18:0 cannot be efficiently incorporated into fish tissues^31^. This is verified by the present result, that is, the C18:0 contents in liver and muscle were not significantly influenced by dietary C18:0 contents. Besides MUFA, n-3 LC-PUFAs were also significantly influenced by ARA. The contents of EPA, DPA, and DHA in muscle were reversely related to the ARA contents. This phenomenon has also been observed in previous studies^6,58,59^. ARA may inhibit Δ6 and/or Δ5 desaturase activity and impair n-3 LC-PUFA biosynthesis by feedback mechanisms.

Besides lipid metabolism, in the present study another physiological process which was significantly influenced by dietary ARA could be cell cycle progression. Transcription of Cyclin B1 and Cdc2 (CDK1), which activate mitosis by forming Cdc2-cyclin B1 complex, as well as AURKA, which regulates spindle assembly, were all down-regulated by moderate levels of dietary ARA (0.22~0.60%). Gene expression of Cyclin I, of which the function remains obscure but has been assumed to be linked to angiogenesis and cell proliferation^60–62^, was also down-regulated by moderate levels of dietary ARA (0.37% and 0.60%). Similar regulating patterns were observed regarding the effects of moderate levels of dietary ARA on hepatic protein concentration of Cyclin B1, Cdc2, AURKA, and Cyclin I. This indicated that at this specific growth stage of Japanese seabass, moderate levels of dietary ARA tended to inhibit the cell cycle progression in liver. The inhibition of Cyclin B1 and subsequent CDK1 activation in liver by ARA-enriched fungal oil has also been observed in a feeding study with mouse^20^. In Jurkat cells, ARA treatment down-regulated the gene expressions of Cyclins A and B1 and cell division cycle 25 homolog A (Cdc25A)^21^. In many *in vitro* studies, it has been observed that ARA or ARA-derived peroxidation products inhibited cell cycle progression and promoted apoptosis, and thus may have the potential of killing cancer cells^22–24^. However, contradictory results also widely existed. High levels of ARA have been showed to stimulate Cyclin E expression, promote faster G1/S transition, and thus promote cell proliferation in human breast cancer tissues^25^. Metabolites of ARA peroxidation have also been reported to have mitogenic effects and promote cell proliferation^26,27^. In mouse embryonic stem cells, ARA up-regulated short time-period hypoxia-induced G1 phase Cyclins D1 and E, and CDK 2 and 4, through the cooperation of PI3K/Akt/mTOR, MAPK and cPLA2-mediated signal pathways^28^. Contradictory results even existed in the present study. Hepatic transcription and protein concentration of MYC, which could drive cell proliferation via cyclins, was up-regulated by moderate levels of dietary ARA. The discrepancies in the regulatory effects of ARA on cell cycle progression could be mainly related to the ARA does and cell type. More studies are needed to elucidate the precise mechanisms involved, especially those in fish. Moreover, inconsistent results between gene expression level and protein concentration level were also observed regarding the effects dietary ARA on AURKA and Cdc20, which regulates spindle assembly checkpoint. Dietary ARA significantly affected Cdc20 at the gene expression level but not at the protein concentration level. Compared to the control diet, high levels (1.38% and 2.32%) of dietary ARA decreased the hepatic gene expression of AURKA but did not affected its protein concentration. This confirmed the complicated effects of high ARA levels. Since ARA and its metabolites could act as important intracellular signaling messengers, supplementary *in vitro* studies in future may help better elucidating the dose-dependent regulatory effects of ARA in fish.

Based on the present results, it was difficult to estimate whether the inhibition of liver cell cycle progression was related to the reduction of liver lipid accumulation by dietary ARA. The down-regulation of PFK-2 gene expression by ARA may contribute to the inhibition of cell cycle progression-related gene expressions by ARA, since PFK-2 has been observed to promote cell proliferation by regulating glycolysis or via CDK1-mediated phosphorylation of p27^63,64^. Other energetic links may also exist between the effects of ARA on lipid metabolism and those on cell cycle, but no direct evidence was observed from the present results. Nevertheless, both inhibited cell cycle progression and reduced lipid accumulation in liver may explain the reduction of hepatosmatic index by dietary ARA observed in our previous studies with Japanese seabass^6^. Additionally, the effects of dietary ARA on cell cycle progression-related gene expression must be related to the regulation of fish gonadal development by ARA. In our previous studies with broodstock of another marine species, *C. semilaevis*, as well as in previous fish studies by other workers, dietary ARA has been demonstrated to be able to regulate the gonadal development and the consequent reproductive performances^9,40,65,66^. Investigation on the regulation of cell cycle progression by dietary ARA will help better understanding the mechanisms involved in the effects of dietary ARA on fish gonadal development and the final reproductive performances.

In conclusion, under the present experimental conditions moderate levels (0.37~0.60%) of ARA in diets for Japanese seabass reduced hepatic lipid accumulation via regulation on both lipogenesis and fatty acid β-oxidation, and tended to inhibit cell cycle progression in the liver. The regulatory effects of ARA were sensitive to the ARA doses, and more studies are needed to elucidate the mechanisms involved.

## Methods

### Ethics statement

The present experimental procedures were carried out in strict accordance with the regulations of the Guide for the Use of Experimental Animals of Yellow Sea Fisheries Research Institute. All animal care and use procedures were approved by the Animal Care and Use Committee of Yellow Sea Fisheries Research Institute. Before handling and sacrifice, experimental fish were anesthetized with eugenol, and all efforts were made to minimize fish suffering.

### Experimental diets and feeding trial

Six diets with similar proximate compositions but graded ARA contents were used in this experiment (see Supplementary Table S4 for the detailed formulation). The control diet was formulated using fish meal, soybean meal, and wheat meal as the protein sources, and soy lecithin and tristearin as the lipid sources. An ARA enriched oil (ARA concentration, 46% of total fatty acids (TFA); in the form of triglyceride) was supplemented to the control diet, replacing tristearin, to formulate five diets with graded ARA levels. The ARA concentration in the six diets was 0.26%, 1.24%, 3.04%, 5.97%, 11.44%, and 22.86% of total fatty acids respectively (see Supplementary Table S5 for the detailed fatty acid profile), and the ARA concentrations relative to dietary dry matter were 0.05%, 0.22%, 0.37%, 0.60%, 1.38%, and 2.32% respectively. The corresponding diets were designated as ARA-0.05, ARA-0.22, ARA-0.37, ARA-0.60, ARA-1.38, and ARA-2.32 respectively. The diets were made, packed and stored following the common procedures in our laboratory^67^.

A 12-week feeding trial with juvenile Japanese seabass (average initial body weight, 207.16 g) was conducted. The feeding trial was conducted in sea floating net cages. Each diet was randomly assigned to triplicate cages, each cage stocked with 30 fish. The detailed feeding procedures were according to our previous studies^6^. At the end of the feeding trial, the fish were fasted for 24 h before harvest. Five whole fish and tissue samples from ten fish from each cage were collected, frozen with liquid nitrogen, and stored at −80 °C before usage.

### RNA isolation, cDNA library construction and Illumina sequencing

Total RNA in liver samples was isolated using RNAiso Plus (TaKaRa Biotechnology (Dalian) Co., Ltd., Dalian, China). RNA degradation and contamination was monitored on 1% agarose gels. RNA purity was checked using the Nano Photometer^®^ spectrophotometer (IMPLEN, Westlake Village, CA, USA). RNA concentration was measured using Qubit^®^ RNA Assay Kit in Qubit^®^ 2.0 Flurometer (Life Technologies, Carlsbad, CA, USA). RNA integrity was assessed using the RNA Nano 6000 Assay Kit of the Bioanalyzer 2100 system (Agilent Technologies, Santa Clara, CA, USA).

A total amount of 3 µg RNA per sample was used as input material for the RNA sample preparations and all samples had RIN values >8. Five individual samples from the same experimental cage were pooled in equal amounts to obtain a pooling sample for this replicate cage. Six pooling samples were then used to prepare 6 separate Illumina sequencing libraries (three biological replicates for each dietary group).

cDNA libraries were generated using Illumina TruSeq™ RNA Sample Preparation Kit (Illumia, San Diego, CA, USA) following manufacturer’s recommendations and index codes were added to attribute sequences to each sample. Briefly, mRNA was purified from total RNA using poly-T oligo-attached magnetic beads. Fragmentation was carried out using divalent cations under elevated temperature in NEBNext First Strand Synthesis Reaction Buffer (5×). First strand cDNA was synthesized using random hexamer primer and M-MuLV Reverse Transcriptase (RNase H^−^). Second strand cDNA synthesis was subsequently performed using DNA Polymerase I and RNase H. Remaining overhangs were converted into blunt ends via exonuclease/polymerase activities. After adenylation of 3′ ends of DNA fragments, NEBNext Adaptor with hairpin loop structure was ligated to prepare for hybridization. In order to select cDNA fragments of 150~200 bp in length, the library fragments were purified with AMPure XP system (Beckman Coulter, Beverly, MA, USA). Then 3 μl USER Enzyme (NEB, Ipswich, MA, USA) was used with size-selected, adaptor-ligated cDNA at 37 °C for 15 min followed by 5 min at 95 °C before PCR. Then PCR was performed with Phusion High-Fidelity DNA polymerase, Universal PCR primers and Index (X) Primer. At last, PCR products were purified (AMPure XP system) and library quality was assessed on the Agilent Bioanalyzer 2100 system.

The clustering of the index-coded samples was performed on a cBot Cluster Generation System using TruSeq PE Cluster Kit v3-cBot-HS (Illumina) according to the manufacturer’s instructions. After cluster generation, the library preparations were sequenced on an Illumina Hiseq2000 platform (Illumina, Inc., San Diego, CA, USA) and 150 bp paired-end reads were generated.

### Bioinformatic analysis

Raw data (raw reads) in fastq format were firstly processed through in-house perl scripts. Clean data (clean reads) were obtained by removing reads containing adapter, reads containing ploy-N and low quality reads from raw data. All the downstream analyses were based on clean data with high quality.

For transcriptome assembly, the left files (read1 files) from all libraries/samples were pooled into one big left.fq file, and right files (read2 files) into one big right.fq file. Transcriptome assembly was then accomplished based on the left.fq and right.fq using Trinity^68^ with min_kmer_cov set to 2 by default and all other parameters set default. Gene function was annotated based on the following databases: Nr; Nt; Pfam; KOG/COG; Swiss-Prot; KO; and GO. Gene expression levels were estimated by RSEM^69^ for each sample.

Clean data were mapped back onto the assembled transcriptome, and read count for each gene was obtained from the mapping results. Differential expression analysis between two experimental groups (three biological replicates for each group) was performed using the DESeq R package (1.10.1). DESeq provide statistical routines for determining differential expression in digital gene expression data using a model based on the negative binomial distribution. The resulting *P* values were adjusted using the Benjamini and Hochberg’s approach for controlling the false discovery rate. Genes with an adjusted *P*-value < 0.05 found by DESeq were assigned as differentially expressed. KEGG enrichment analysis of the DEGs was performed using KOBAS^70^ software.

### Real-time quantitative polymerase chain reaction (qRT-PCR) validation of Illumina sequencing data

To validate the Illumina sequencing data, twelve differentially expressed genes were selected for quantitative RT-PCR analysis, using the same RNA samples for the transcriptome profiling. Quantitative RT-PCR analysis of fifteen more genes linked to lipid metabolism was also conducted. Specific primers were designed based on the Illumina sequencing data (Table 3). For DGAT and AURKA, of which the sequences from transcriptome data failed to supply high quality specific primers, internal sequence fragments were cloned by the authors, and specific primers were designed based on the cloned sequence fragments (Table 3, GenBank accession No. MF417632 and MF417633 respectively). β-actin (GenBank accession No. HE577671.1) was used as the reference gene according to our previous screening^33^. The real-time PCR was carried out with SYBR Green Real-time PCR Master Mix (TaKaRa Biotechnology (Dalian) Co., Ltd., Dalian, China) in a quantitative thermal cycler (Mastercycler eprealplex, Eppendorf, German). The detailed program was similar with Xu *et al*.^32^. The mRNA expression levels were studied by qRT-PCR method: 2^−ΔΔCT ^^71^. All the qRT-PCR measurements were conducted with liver samples from all the six experimental groups.

**Table 3:** Primers used in this work.

Gene	Forward primer sequences (5′-3′)	Reverse primer sequences (5′-3′)
*Real-time quantitative PCR*		
SCD1	TACTCCGAGACAGACGCAGAC	GCCACAGCCAAGGATTCAC
ApoE	TGGCAGAGCTGGCTACATACA	TTGCGGACATCGTCAGAGTT
CPT-1α	GCTGTTGCCACGGGAGATAG	CTGCTCAGTGTCATCAAGGGTT
FABP1	TGAACTCATCCAGAAAGGCAA	CCGTCACGCTGAACCACA
PEPCK	CCTGCTGCGAGTCCCTGA	TACTTCCTGACCTCCTCCACC
GS	CTTGAGGAGTGGGACGAGGA	GAGGTCAGACGGGCTTTGGT
LPL	TGTGTCCAAGTTCTCCCTGCG	CCAGCCATGTATCACAATGAAGC
FAS	TCGTCATGGCTATGAGGGAGAT	GTTGAGTGGCGTCACTGTGG
ME	CTTGCAGAGCAGGTGACAGATAA	GGCAGGAAGGAGTCGTAGTGG
6GPD	CAGGGACGAGGAGGCTACTT	CACGTCTGACATGGACACTGG
SREBP1	TGCTATCGGTTCTAACATGGCTAC	AGTGCTCAACAGTCAGATACAGTC
DGAT	CTGAGCCATCATTCCCATAAAG	GTTCACAAGTGGTGCCTGAGAC
GPAT	AATGAAACGCCAACCCTGC	GTGTAGTGCTCCTGAAATCTATCCT
ACO1	CACTGGTAGATGCCTTTGACG	CATGGACCTCTGTGGAGTTTAG
PPARα2	TTCCAGCTGGCAGAGAGGACGC	CACCCCACAGCCGGAACCACCT
PPARβ	GCCAGGGAAAGTGAAGATGAGG	AGCGAGGGCGACGAGGTATG
PPARγ	AGAGCGAAGAGCACCTGACC	GCTTGGAGAACACGGGACT
ApoA1	GAGCGTCTGGAGAGTCTGAGG	ACGGAAGCAGCGATGAGC
ApoA4	AGAAGAGCGAGGAACTCAAGGC	GAGGTCGTCGGTGTAGGGTC
ApoB100	TTTTCGCTCTGGGACTCAT	GCTTGCTGGGCTTGTATTT
PFK-2	CAAAGCCGAATCGTCTACTACC	CATCAGGCTTCTGGCATACTCT
Cyclin B1	AAACCCAGTCCCCAAGAAAC	GTCGGTCACGTAGGCAAAGT
Cdc2	ACCCCAGAACCTCCTGATTGAC	AGGCTTCTTGGTGGCGAGTT
MYC	GACGGTGGACAGGCGAAAG	GGCGTAGTTGTGCTGATGGATG
Cdc20	AAGGCTTTGGGCTGGTGTC	TCTGTCTTCGTGTCCGTTGAG
AURKA	TCACCTCAGGCACCCCAACA	CCTCAGGAAAAGATCCACAGCG
Cyclin I	ATAGGCTCTGTTAAAGACCTGGTTGC	GGGCGTGGAATATGTGAATGAAGT
β-actin	CAACTGGGATGACATGGAGAAG	TTGGCTTTGGGGTTCAGG
*Reverse transcription PCR for cloning internal sequence fragments*		
DGAT	CTACCCAGGMAACCTCACMC	AAGCGRCCCACGAACCAAGC
AURKA	GAGCCTGGARAAYTTTGASATTGG	CATCTGYCAGYTCCATGATGTAHGT

SCD1, stearoyl-CoA desaturase 1; ApoE, apolipoprotein E; CPT-1α, carnitine O-palmitoyltransferase-1α; FABP1, fatty acid-binding proteins 1; PEPCK, phosphoenolpyruvate carboxykinase; GS, glycogen synthase; LPL, lipoprotein lipase; FAS, fatty acid synthetase; ME, malic enzyme; 6GPD, glucose-6-phosphate dehydrogenase; SREBP1, sterol-regulatory element binding proteins 1; DGAT, diacylglycerol acyl transferase; GPAT, glycerol-3-phosphate acyltransferase; ACO1, acyl-CoA oxidase 1; PPAR, peroxisome proliferator-activated receptor; PFK-2,6-phosphofructo-2-kinase (PFK-2); Cyclin B1, G2/mitotic-specific cyclin-B1; Cdc2, cyclin-dependent kinase 1 (CDK1); MYC, myc proto-oncogene protein; Cdc20, Cell division cycle protein 20; AURKA, aurora kinase A.

### Analysis of hepatic protein concentration with ELISA methods

All the assay was done with commercial ELISA kits for fish (Shanghai Enzyme-linked Biotechnology Co., Ltd., Shanghai, China) according to the user’s manuals. Briefly, one-step double antibody sandwich method was used^72^. Mouse anti-zebrafish monoclonal antibodies and rabbit anti-zebrafish polyclonal antibodies were commercially supplied by Shanghai Enzyme-linked Biotechnology Co., Ltd., Shanghai, China. Liver samples (10 mg) were homogenized in PBS buffer solution (100 μL), centrifuged (2500 r min^−1^, for 20 min), and then the supernatant was collected for following assays. Horse radish peroxidase (HRP) labelled on antibodies and tetramethylbenzidine (TMB) were used to generate solution color for spectrophotometry. Standard curves were plotted based on optical density of protein standard assays, and experimental samples were assayed at the same time. The results was expressed as ng or μg g^−1^ liver tissue. The inter- and intra-assay CV for the assays were <10% and <15%, respectively.

### Analysis of proximate composition, lipid concentration and fatty acid composition

Proximate composition analysis of experimental diets was performed by the standard methods of Association of Official Analytical Chemists (AOAC). For lipid analyses of whole fish body, liver, and muscle, extraction was done with chloroform-methanol method according to Folch *et al*.^73^. The fatty acid compositions of diet and fish tissues were analyzed via a gas chromatograph, using a flame ionization detector (FID). Fatty acids in freeze-dried samples were esterified first with KOH-methanol and then with HCL-methanol, in 72 °C water bath. Fatty acid methyl esters were extracted with hexane and then separated via gas chromatography (HP6890, Agilent Technologies, Santa Clara, CA, USA) with a fused silica capillary column (007-CW, Hewlett Packard, Palo Alto, CA, USA). The column temperature was programmed to rise from 150 °C up to 200 °C at a rate of 15 °C min^−1^, and then from 200 °C to 250 °C at a rate of 2 °C min^−1^. Both the injector and detector temperatures were 250 °C. Results are expressed as the percentage of each fatty acid with respect to total fatty acids.

### Statistical analysis

The data of qRT-PCR, lipid content and fatty acid composition were subjected to one-way analysis of variance in SPSS 16.0 (SPSS Inc., Chicago, USA) for Windows. All percentage data were arcsine transformed prior to analysis. Differences between means were tested by Tukey’s multiple range test. The level of significance was chosen at *P* < 0.05 and the results were presented as means ± standard error.

### Data availability

The datasets generated during and/or analysed during the current study are available from the corresponding author on reasonable request.

## Electronic supplementary material

Supplementary Information
