# Supplementary material for: Moderate levels of dietary arachidonic acid reduced lipid accumulation and tended to inhibit cell cycle progression in the liver of Japanese seabass Lateolabrax japonicus
Source: Sci Rep. 2018 Jul 16;8:10682. doi: 10.1038/s41598-018-28867-z (PMC6048150; doi:10.1038/s41598-018-28867-z)
Supplement: Supplementary file 1 — Supplementary Information [file 41598_2018_28867_MOESM1_ESM.pdf]

**SUPPLEMENTARY INFORMATION:**

# Moderate levels of dietary arachidonic acid reduced lipid accumulation and tended to inhibit cell cycle progression in the liver of Japanese seabass *Lateolabrax japonicus*

Houguo Xu[1], Chengqiang Wang[1], Yuanqin Zhang[1], Yuliang Wei[1], Mengqing Liang*[1,2]

[1]Yellow Sea Fisheries Research Institute, Chinese Academy of Fishery Sciences, 106 Nanjing Road, Qingdao, 266071 Shandong, China

[2]Laboratory for Marine Fisheries Science and Food Production Processes, Qingdao National Laboratory for Marine Science and Technology, 1 Wenhai Road, Qingdao, 266237 Shandong, China

*__Corresponding author:__ Mengqing Liang, E-mail: liangmq@ysfri.ac.cn

**Supplementary Table S1. Clean data and quality control.**

| Sample | Raw Reads | Clean Reads | Clean Bases | Error (%) | Q20 (%) | Q30 (%) | GC Content (%) |
|---|---|---|---|---|---|---|---|
| ARA-0.05_1 | 61,590,114 | 57,285,590 | 8.59 G | 0.02 | 96.64 | 90.87 | 48.57 |
| ARA-0.05_2 | 57,541,780 | 53,078,734 | 7.96 G | 0.02 | 96.47 | 90.49 | 49.65 |
| ARA-0.05_3 | 58,486,918 | 45,451,256 | 6.82 G | 0.02 | 96.82 | 90.23 | 49.70 |
| ARA-0.37_1 | 62,357,214 | 53,071,432 | 7.96 G | 0.02 | 97.08 | 91.43 | 48.42 |
| ARA-0.37_2 | 58,575,334 | 52,696,304 | 7.90 G | 0.02 | 96.77 | 90.94 | 49.31 |
| ARA-0.37_3 | 61,039,602 | 59,417,542 | 8.91 G | 0.02 | 95.74 | 89.54 | 49.10 |

Q20 and Q30: the sequencing error rate 0.01 and 0.001 respectively.

**Supplementary Table S2. Length distribution of transcripts and unigenes.**

| Transcript length interval | 200-500 bp | 500-1k bp | 1k-2k bp | >2k bp | Total |
|---|---|---|---|---|---|
| Number of transcripts | 145,811 | 42,334 | 33,836 | 39,966 | 261,947 |
| Number of unigenes | 133,117 | 29,326 | 15,570 | 13,844 | 191,857 |

**Supplementary Table S3. Annotation of the unigenes.**

| Annotated in database | Number of unigenes | Percentage (%) |
|---|---|---|
| Annotated in NR | 46,692 | 24.33 |
| Annotated in NT | 66,582 | 34.70 |
| Annotated in KO | 25,420 | 13.24 |
| Annotated in SwissProt | 38,541 | 20.08 |
| Annotated in PFAM | 37,741 | 19.67 |
| Annotated in GO | 37,959 | 19.78 |
| Annotated in KOG | 21,546 | 11.23 |
| Annotated in all Databases | 12,801 | 6.67 |
| Annotated in at least one Database | 81,636 | 42.55 |
| Total Unigenes | 191,857 | 100 |

**Supplementary Table S4. Formulation and proximate composition of the experiment diets (% dry matter).**

| Ingredients | Dietary ARA level (% dry matter) | | | | | |
|---|---|---|---|---|---|---|
| | 0.05 | 0.22 | 0.37 | 0.60 | 1.38 | 2.32 |
| Fish meal | 15.00 | 15.00 | 15.00 | 15.00 | 15.00 | 15.00 |
| Soybean meal | 20.00 | 20.00 | 20.00 | 20.00 | 20.00 | 20.00 |
| Wheat meal | 30.85 | 30.85 | 30.85 | 30.85 | 30.85 | 30.85 |
| Casein | 16.00 | 16.00 | 16.00 | 16.00 | 16.00 | 16.00 |
| Gelatin | 4.00 | 4.00 | 4.00 | 4.00 | 4.00 | 4.00 |
| Mineral premix[a] | 2.00 | 2.00 | 2.00 | 2.00 | 2.00 | 2.00 |
| Vitamin premix[b] | 2.00 | 2.00 | 2.00 | 2.00 | 2.00 | 2.00 |
| Attractant[c] | 0.30 | 0.30 | 0.30 | 0.30 | 0.30 | 0.30 |
| Mold inhibitor[d] | 0.10 | 0.10 | 0.10 | 0.10 | 0.10 | 0.10 |
| Ethoxyquin | 0.05 | 0.05 | 0.05 | 0.05 | 0.05 | 0.05 |
| Soy lecithin | 1.20 | 1.20 | 1.20 | 1.20 | 1.20 | 1.20 |
| DHA enriched oil[e] | 1.50 | 1.50 | 1.50 | 1.50 | 1.50 | 1.50 |
| ARA enriched oil[f] | 0.00 | 0.35 | 0.71 | 1.45 | 2.91 | 5.84 |
| Tristearin[g] | 7.00 | 6.65 | 6.29 | 5.55 | 4.09 | 1.16 |
| *Proximate composition* | | | | | | |
| Crude protein | 43.64 | 43.53 | 44.40 | 44.36 | 43.39 | 44.13 |
| Crude lipid | 11.32 | 11.07 | 11.09 | 11.24 | 11.50 | 11.22 |
| Ash | 6.89 | 6.94 | 6.81 | 6.98 | 6.92 | 6.83 |

[a]Mineral premix (mg or g/kg diet): $MgSO_4 \cdot 7H_2O$, 1200 mg; $CuSO_4 \cdot 5H_2O$, 10 mg; $ZnSO_4 \cdot H_2O$, 50 mg; $FeSO_4 \cdot H_2O$, 80 mg; $MnSO_4 \cdot H_2O$, 45 mg; $CoCl_2 \cdot 6H_2O$ (1%), 50 mg; $NaSeSO_3 \cdot 5H_2O$ (1%), 20mg; $Ca(IO_3)_2 \cdot 6H_2O$ (1%), 60mg; $CaH_2PO_4 \cdot H_2O$, 10g; zoelite, 8.485 g.

[b]Vitamin premix (mg or g/kg diet): thiamin 25 mg; riboflavin, 45 mg; pyridoxine HCl, 20 mg; vitamin $B_{12}$ (1%), 10 mg; vitamin $K_3$, 10 mg; inositol, 800 mg; pantothenic acid, 60 mg; niacin, 200 mg; folic acid, 20 mg; biotin (2%), 60 mg; retinol acetate, 32 mg; cholecalciferol, 5 mg; alpha-tocopherol (50%), 240 mg; ascorbic acid, 5 g; choline chloride (50%), 5 g; wheat middling, 4.47 g.

[c]Attractant: glycine and betaine.

[d]Mold inhibitor: contained 50% calcium propionic acid and 50% fumaric acid.

[e]DHA enriched oil: DHA content, 43% of total fatty acid; in the form of triglyceride; Jiangsu Tiankai Biotechnology Co., Ltd., Nanjing, China.

[f]ARA enriched oil: ARA content, 46% of total fatty acid; in the form of triglyceride; Jiangsu

Tiankai Biotechnology Co., Ltd., Nanjing, China.

[g]Tristearin: HUDONG article of everyday use Co., Ltd., Jiaxing, China.

**Supplementary Table S5. Fatty acid profiles of experimental diets (% total fatty acid).**

| Fatty acid | ARA-0.05 | ARA-0.22 | ARA-0.37 | ARA-0.60 | ARA-1.38 | ARA-2.32 |
|---|---|---|---|---|---|---|
| C14:0 | 3.50 | 3.61 | 3.61 | 3.50 | 3.00 | 2.38 |
| C16:0 | 26.62 | 27.12 | 26.62 | 25.91 | 22.38 | 17.35 |
| C18:0 | 44.46 | 44.10 | 41.84 | 38.76 | 29.18 | 12.88 |
| ΣSFA | 74.58 | 74.83 | 72.07 | 68.17 | 54.56 | 32.61 |
| C16:1n-7 | 0.79 | 0.81 | 0.84 | 0.88 | 0.87 | 0.92 |
| C18:1n-9 | 3.87 | 3.42 | 3.69 | 3.85 | 5.16 | 6.72 |
| ∑MUFA | 4.66 | 4.23 | 4.53 | 4.73 | 6.03 | 7.64 |
| C18:2n-6 | 7.32 | 8.05 | 8.21 | 8.72 | 9.71 | 12.19 |
| C20:4n-6 | 0.26 | 1.24 | 3.04 | 5.97 | 11.44 | 22.86 |
| ∑n-6 PUFA | 7.32 | 9.29 | 11.25 | 14.69 | 21.15 | 35.05 |
| C18:3n-3 | 0.70 | 0.76 | 0.81 | 0.80 | 0.85 | 0.92 |
| C20:5n-3 | 1.37 | 1.44 | 1.49 | 1.49 | 1.65 | 1.57 |
| C22:6n-3 | 6.26 | 6.39 | 6.59 | 6.63 | 6.60 | 6.66 |
| ∑n-3 PUFA | 8.33 | 8.59 | 8.89 | 8.92 | 9.10 | 9.15 |
| ∑PUFA | 15.65 | 17.88 | 20.14 | 23.61 | 30.25 | 44.20 |
| ΣSFA/ΣPUFA | 4.77 | 4.19 | 3.58 | 2.89 | 1.80 | 0.74 |
| ∑n-3/∑n-6 | 1.14 | 0.92 | 0.79 | 0.61 | 0.43 | 0.26 |

SFA, saturated fatty acid; MUFA, monounsaturated fatty acid; PUFA, polyunsaturated fatty acid.

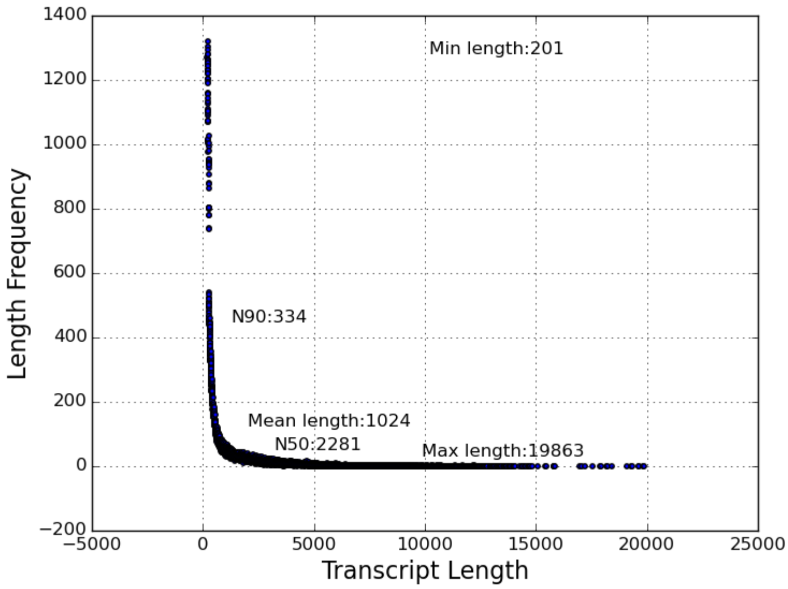


**Supplementary Fig. S1. Length distribution of transcripts.** N50 (N90): length of the trancript, longer than which the cumulative length of transcripts acounts for 50% (90%) of the total cumulative length.

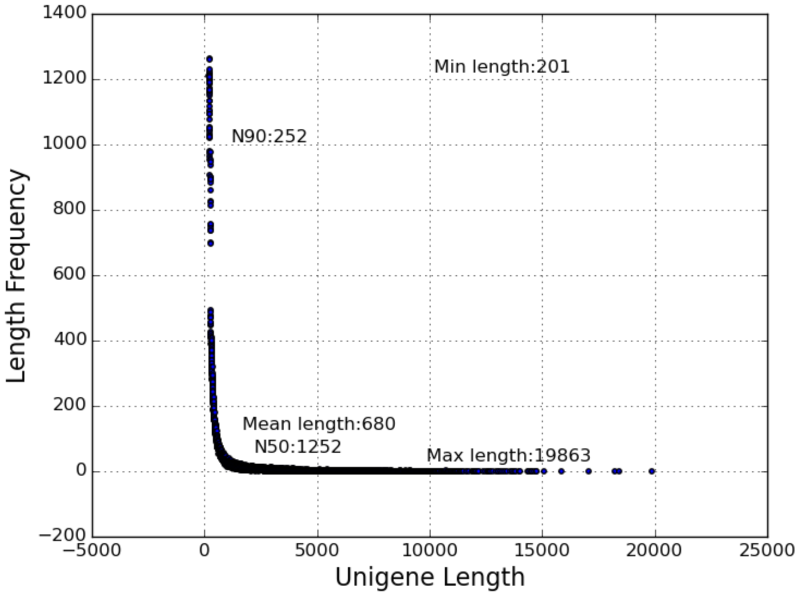


**Supplementary Fig. S2. Length distribution of unigenes.** N50 (N90): length of the unigene, longer than which the cumulative length of unigenes acounts for 50% (90%) of the total cumulative length.

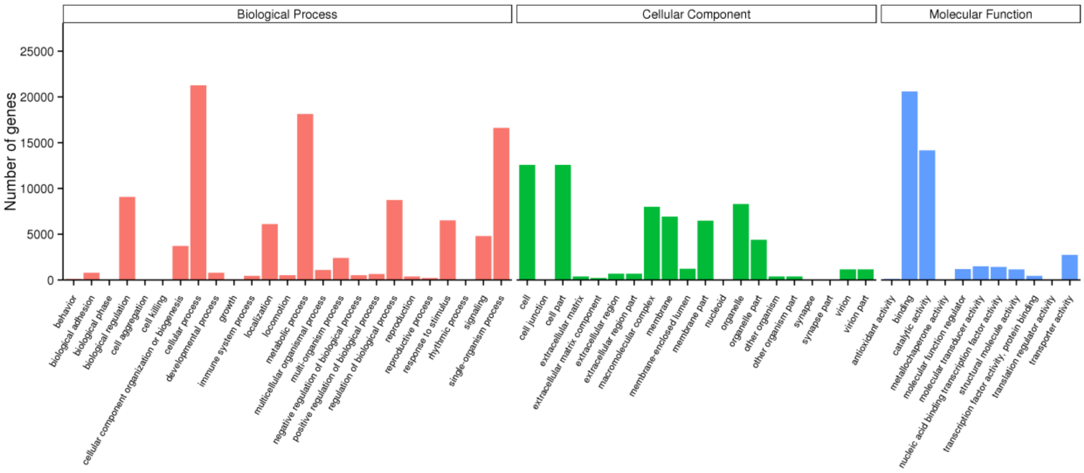


**Supplementary Fig. S3. Gene function classification (GO) of unigenes.**

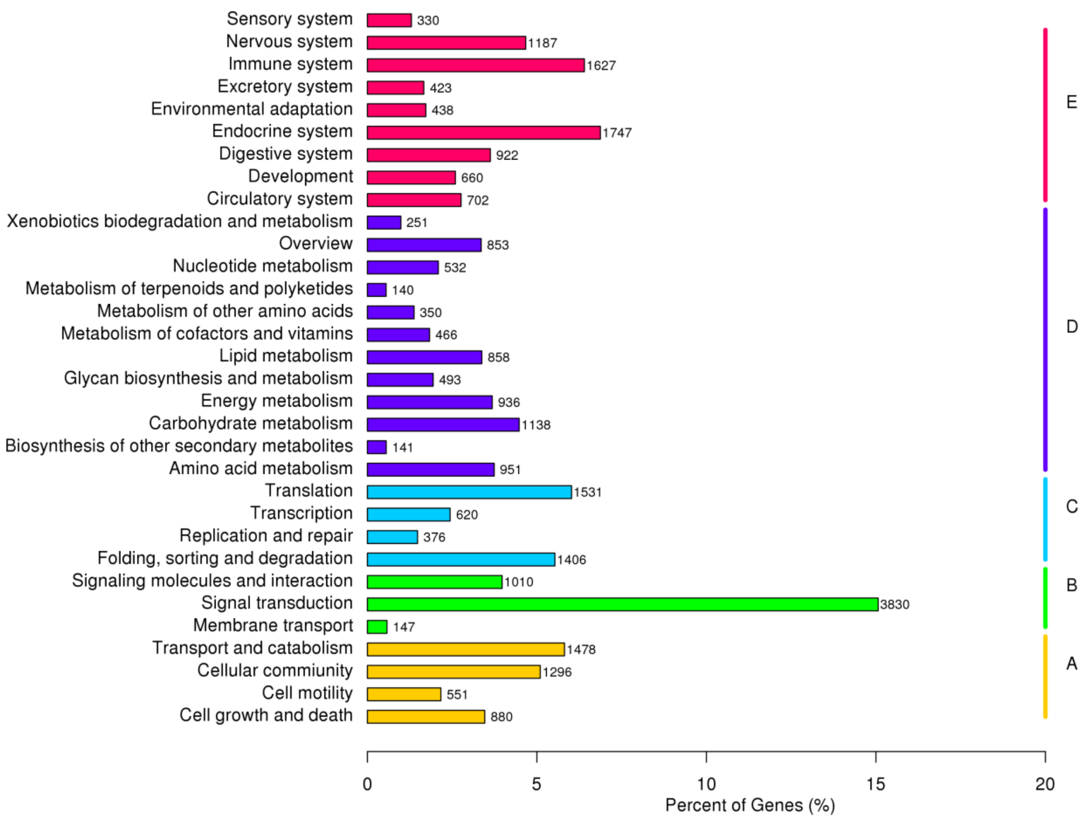


**Supplementary Fig. S4. KEGG classification of unigenes.** A: cellular processes; B: environmental information processing; C: genetic information processing; D: metabolism; E: organismal systems.

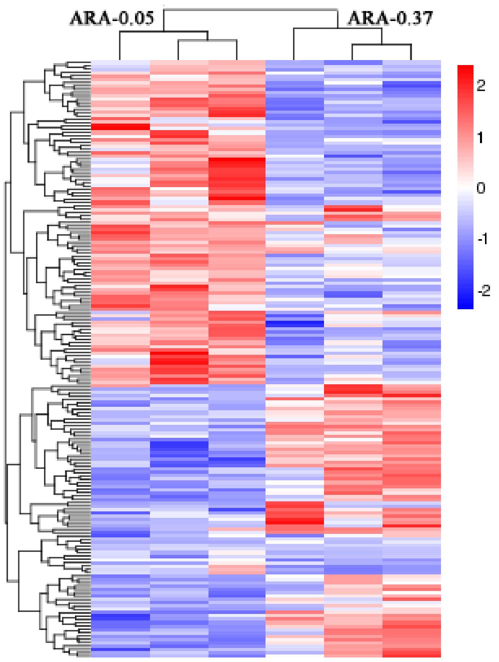


**Supplementary Fig. S5. Cluster analysis of differentially expressed genes.**
